# General practitioners’ perspectives regarding early developmental surveillance for autism within the australian primary healthcare setting: a qualitative study

**DOI:** 10.1186/s12875-023-02121-6

**Published:** 2023-08-10

**Authors:** Josephine Barbaro, Teresa Winata, Melissa Gilbert, Radhika Nair, Feroza Khan, Abbie Lucien, Raisa Islam, Anne Masi, Antonio Mendoza Diaz, Cheryl Dissanayake, Lisa Karlov, Joseph Descallar, John Eastwood, Iqbal Hasan, Bin Jalaludin, Jane Kohlhoff, Siaw-Teng Liaw, Raghu Lingam, Natalie Ong, Chun Wah Michael Tam, Sue Woolfenden, Valsamma Eapen

**Affiliations:** 1 Olga Tennison Autism Research Centre, School of Psychology and Public Health, La Trobe University, Bundoora, VIC Australia; 2 Cooperative Research Centre for Living with Autism (Autism CRC), The University of Queensland, Indooroopilly, QLD Australia; 3 Discipline of Psychiatry and Mental Health, School of Clinical Medicine, University of New South Wales, Sydney, NSW Australia; 4 Academic Unit of Infant, Child and Adolescent Psychiatry, South Western Sydney Local Health District, Sydney, NSW Australia; 5 Ingham Institute for Applied Medical Research, Liverpool, NSW Australia; 6 Faculty of Medicine and Health, School of Medicine, University of Sydney, Sydney, NSW Australia; 7 Faculty of Medicine, School of Women and Children’s Health, University of New South Wales, Sydney, NSW Australia; 8 Faculty of Medicine, School of Population Health, University of New South Wales, Sydney, NSW Australia; 9 Population Child Health Research Group, Faculty of Medicine, School of Women’s and Children’s Health, University of New South Wales, Sydney, NSW Australia; 10 Children’s Hospital Westmead Clinical School, University of Sydney, Sydney, NSW Australia; 11 Primary and Integrated Care Unit, South Western Sydney Local Health District, Liverpool, NSW Australia

**Keywords:** Families, Healthcare Professional, Primary Healthcare, Growth and development, Autism, Screening

## Abstract

**Background:**

Significant challenges remain in the early identification of child developmental disabilities in the community. Implementing supports and services early in the life course has been shown to promote positive developmental outcomes for children at high likelihood of developmental disabilities, including autism. As part of a cluster randomised controlled trial, this study seeks to examine and compare the perspectives and experiences of Australian general practitioners (GPs) in relation to a digital developmental surveillance program for autism and usual care pathway, in general practice clinics.

**Methods:**

A qualitative research methodology with semi-structured interviews and thematic inductive analysis underpinned by grounded theory was utilised. All GPs from South Western Sydney (NSW) and Melbourne (Victoria) who participated in the main program (“GP Surveillance for Autism”) were invited to the interview. GPs who provided consent were interviewed either over online or in-person meeting. Interviews were audio-recorded, transcribed, and coded using NVivo12 software. Inductive interpretive approach was adopted and data were analysed thematically.

**Results:**

Twenty-three GPs across the two sites (NSW: n = 11; Victoria: n = 12) agreed to be interviewed; data saturation had reached following this number of participants. Inductive thematic coding and analysis yielded eight major themes and highlighted common enablers such as the role of GPs in early identification and subsequent supports, enhanced communication between clinicians/professionals, relationship-building with patients, and having standardised screening tools. Specific facilitators to the feasibility and acceptability of a digital screening program for the early identification of developmental disabilities, including the early signs of autism, and encouraging research and education for GPs. However, several practical and socioeconomic barriers were identified, in addition to limited knowledge and uptake of child developmental screening tools as well as COVID-19 lockdown impacts. Common and specific recommendations involve supporting GPs in developmental/paediatrics training, streamlined screening process, and funding and resources in the primary healthcare services.

**Conclusions:**

The study highlighted the need for practice and policy changes, including further training of GPs alongside sufficient time to complete developmental checks and appropriate financial remuneration through a Medicare billing item. Further research is needed on implementation and scale up of a national surveillance program for early identification of developmental disabilities, including autism.

**Supplementary Information:**

The online version contains supplementary material available at 10.1186/s12875-023-02121-6.

## Background

The early identification of developmental disabilities including autism is vital as it can lead to children accessing appropriate and timely evidence-based supports and services [1]. Children who have undetected developmental disabilities early in life are more likely to develop later health, developmental, learning, and behavioural issues, which in turn can have a negative cumulative effect over the life course [2].

Developmental surveillance offers opportunities to identify children with developmental differences, including the early signs of autism in a systematic way through health and developmental monitoring, thereby facilitating opportunities for early supports and services. Over the past three decades, data have emerged suggesting that developmental disabilities with signs emerging in infancy and toddlerhood have the potential to affect key outcomes in the longitudinal trajectory of these children [3]. Similarly, it has been recognised that the earlier that supports and services are accessed, the better the outcomes for the child [4]. There is also increasing evidence to suggest that early identification, diagnosis, and supports/services are efficacious, cost-effective, and may help reduce the health inequality and disparities needed to break the cycles of intergenerational disadvantage [5–8]. Though some parents/caregivers may have concerns about their child’s development from a very early age, children on the autism spectrum are identified and diagnosed later than children with general developmental delay or intellectual disability [9]. In Australia, the average age of autism diagnosis for children under seven years is 49 months, [10] substantially higher than 24 months – the recognised age at which autism can be reliably diagnosed [11]. Such delays in identification and diagnosis can also result in increased parental stress and significant delays in initiating early supports, which can result in less-than-optimal outcomes over time [12].

In the current study, general practitioners (GPs) were engaged and involved in an early developmental surveillance program for developmental disabilities, including autism, using a digital developmental surveillance framework during opportunistic contacts in the general practice setting [13]. In Australia, GPs are often the first point of contact within the health care system, and they treat and co-ordinate care for all common heath conditions including referral to other specialists. As universal service providers, GPs play a critical role in equitable health care access for children and families. This qualitative study aimed to ascertain, and evaluate participating stakeholders’ (both parents/caregivers and GPs) perspectives of the enablers, barriers, and suggestions for improvements in the implementation of the integrated model of developmental surveillance and referral used in the intervention (Autism Surveillance Pathway [ASP]) arm of the trial, including the uptake of recommendations, service access and satisfaction. A similar study, previously conducted by our team, investigated healthcare professionals’ perceptions of screening tools for the general developmental surveillance program for preschool-aged children in the ‘Watch Me Grow’ (WMG) project [14]. The qualitative findings based on this previous work indicated that there was lack of awareness among parents about the need for child developmental checks, a limited knowledge among health professionals about child developmental screening tools in general, as well as the need for increasing the specificity of these tools to include culturally and linguistically diverse (CALD) parents/caregivers [15, 16]. While the current parental/caregivers’ experiences and perspectives have been reported elsewhere [17], this study has focussed on the feasibility and experience of an integrated early child development surveillance and care pathway in the Australian primary healthcare setting through a GP lens and viewpoints.

**Research aims.** Using qualitative research methodology, this study aimed to:

1. Identify perceptions, barriers, and solutions for GPs when conducting early childhood developmental surveillance including autism surveillance, within the general practice setting,
2. Identify the practice systems required to enable early childhood developmental surveillance activities to be widely adopted in general practice, and.
3. Investigate how GPs perceive their role in early identification and in providing ongoing care for children with developmental disabilities, including autism.

## Methods

### Study context

This article forms one part of the qualitative component of the main program “A multistate trial of an early surveillance program for autism within General Practices in Australia” [13]. The study was established in 2019 as a longitudinal, cluster randomised controlled trial (cRCT) funded by the Cooperative Research Centre for Living with Autism (Autism CRC). It was conducted in 53 GP clinics across the Australian states of NSW and Victoria, with the clinics allocated randomly to either the intervention group (ASP), or the control group (the Surveillance as Usual (SaU) pathway). The overall objective in undertaking the study was to examine and evaluate the implementation of a universal developmental surveillance program, with a particular focus on autism, in the primary care setting [13]. Briefly, parents/caregivers of children aged 18–24 months were invited to participate within general practitioner waiting rooms during opportunistic contacts (e.g., illness, immunisation) with a participating GP. Following completion of informed consent, parents/caregivers completed a brief demographic questionnaire. In the ASP arm, while in the clinic waiting room the parent/caregiver also completed several screening tools: ‘Learn the Signs. Act Early’ (LTSAE), [18] the Parents’ Evaluation of Developmental Status (PEDS), [19] and Quantitative Checklist for Autism in Toddlers (Q-CHAT-10) [20, 21]. During the consultation, the GP administered the online version of the Social Attention and Communication Surveillance tool, implemented online (SACS Online), [22] with GPs using results to determine whether the child had a “high” or “low likelihood” of being autistic. Further, a secondary assessment tool, Ages and Stages Questionnaire-Social Emotional Scale (ASQ-SE) [23] was completed by the parent/caregiver if children were identified as having any concerns from the above measures. For children in the SaU arm, GPs were asked to use their usual practice to determine the child’s likelihood for developmental disabilities. Children in both study arms who were identified as having a “high likelihood” of being autistic were referred to the study team for a standardised diagnostic assessment for autism and other developmental delays/disabilities. Further details on the cRCT methodology, measures and processes can be found in our study protocol [13].

### Participant recruitment and interviews

A total of 53 GPs across South-West Sydney (NSW; n = 30) and Melbourne (Victoria; n = 23) recruited as part of the main study were invited to participate in this interview. A formal email invitation was sent along with a participation information sheet and consent form to the 53 practices. Twenty-three of 53 (43%) GPs across the two sites (NSW: n = 11; Victoria: n = 12) participated in the semi-structured interviews (Table 1 shows the general characteristics of GPs/their practice). A mutually convenient time was arranged with those who provided consent to a 30-minute in-person, Zoom or telephone interview. For GPs in the ASP pathway, additional questions/prompts regarding their experiences with the study tools and procedure were also included. See Table 2 for the general interview questions and Supplementary Table 1 for the full interview guide.

**Table 1:** Characteristics of general practitioners (GPs) enrolled in this qualitative interview study

Features of GPs and its practice	Autism Surveillance Pathway (Treatment Group) GPs	Standard as Usual (Control Group) GPs
Number of GPs (n)	15	8
Gender		
Female Male	8 7	5 3
Type of practice		
Solo Group	2 13	1 7
Billing of practice		
Bulk-billed Private Mixed	5 3 7	4 1 3
Site		
NSW Victoria	9 6	2 6

### Data analysis

Interviews were conducted with participants between 1st May and 26th June 2021 by research staff (TW, AL, MG and RN). All interviews were audio-recorded, transcribed by professional transcription services, and coded using NVivo12 [24]. In utilising an inductive thematic analysis underpinned by grounded theory, the research team has followed a rigorous process to triangulate all collected data by cross-checking and agreeing/disagreeing on the themes and subthemes that have emerged from the qualitative interviews. To do this, transcribed interviews were coded by two researchers (TW, MG). A randomly selected set of interviews were coded by a second set of researchers (FK, AL) and themes were compared. As these researchers were also members of the study team, three random transcripts were also selected and coded by an external reviewer (RI). Any disagreements were resolved through discussions until consensus was reached on themes and subthemes. This system of coding provided an opportunity for identifying a consensus based themes and subthemes of individual experiences and perceptions regarding the feasibility of conducting a developmental surveillance program within the general practice setting [25]. Thematic analysis was undertaken to develop key themes/subthemes relating to GPs’ experiences and perceptions; which was done by inductive coding, [26, 27] allowing data to be organized and used to explore connections between data elements and to develop conceptual items. Once coded, segments of data were then linked in a formal fashion to allow themes to emerge and to determine relationships between different data sets. Data saturation had been reached with the current sample GP size through a strict procedure of pilot interview analysis over multiple iterations/discussions between research members. The study has been reported in line with the Standards for Reporting Qualitative Research (see Supplementary Table 2) [28].

**Table 2:** General interview questions for GPs.

**Main questions for GPs**
Describe your experience of conducting childhood developmental screening/surveillance (DS) in your practice.
Describe what other factors may assist you to conduct DS in your practice?
What barriers did the COVID pandemic and associated changes pose on conducting DS at your practice? Were there any specific enablers that you found helpful in conducting DS?
Describe your experience in managing children whom you (or their parents) identified as having a specific developmental concern.
How do you perceive your role in Early Intervention (EI) for child developmental issues? *FYI The National Disability Insurance Scheme (NDIS) has made available early childhood early intervention (ECEI) services for children aged under seven years of age with a developmental delay or disability.*
Overall, can you describe your role in providing ongoing care for a child with developmental disability (CWDD) within your practice? In particular, we are interested in how you see the extent of your involvement after the children are referred to other services e.g., specialist paediatric or others.
**Additional questions for GPs in the ASP pathway to obtain views on their experiences with the study tools and procedures.**
Can you describe your experience of participating in the ASP subgroup of this study?
Were there any issues; what suggestions do you have to address this to resolve this?
What were your experiences like with completing the SACS Online assessment with children?
What were your experiences like with the parent/caregiver questionnaires – the Q-CHAT-10, ‘Learn the Signs. Act Early’, and the PEDS?
What are your thoughts on using these clinician and parent-completed tools in the future for conducting childhood developmental screening?
Anything else to add?

## Results

**Fig. 1:**
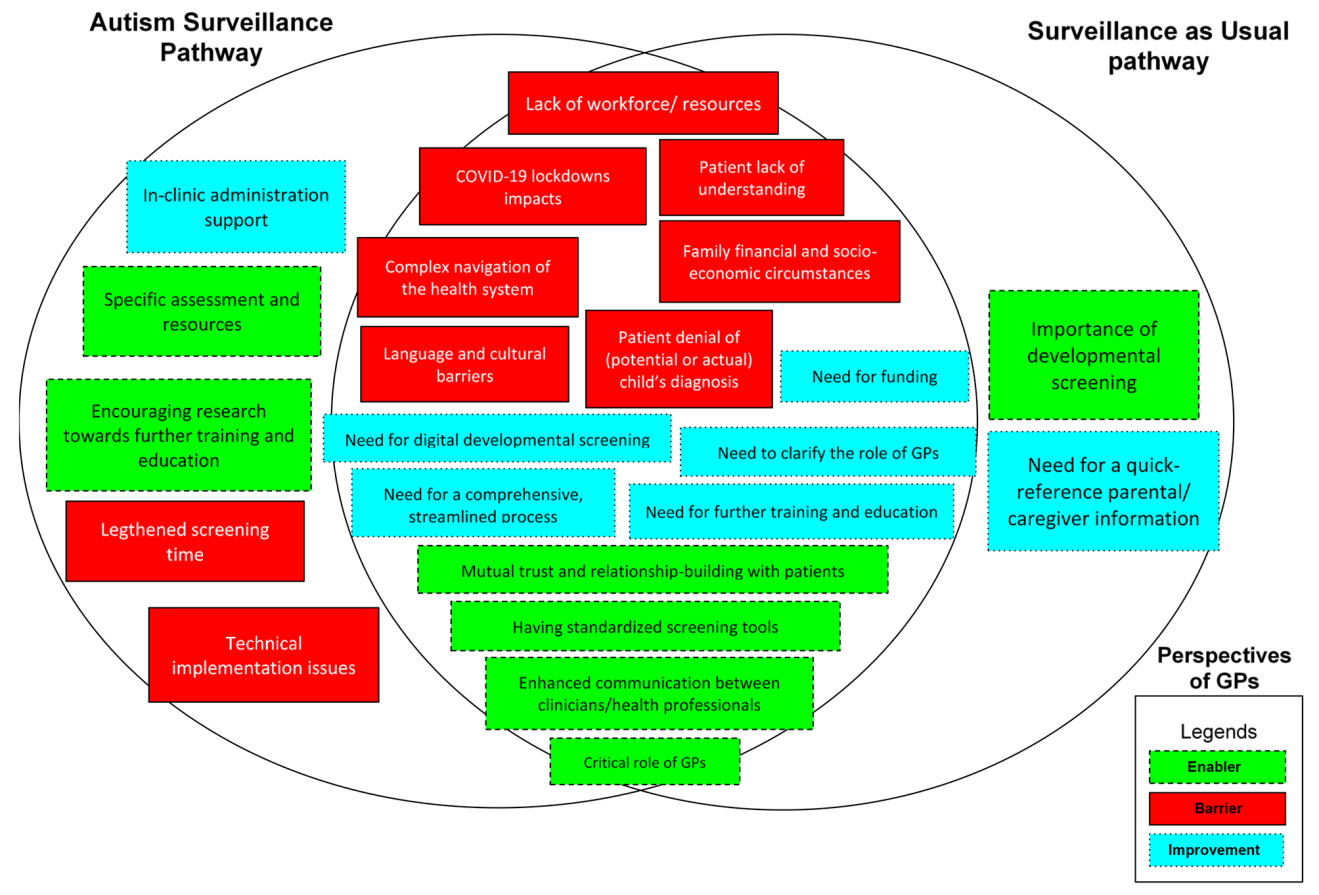
Comparisons of barriers, enablers and suggestive improvements out of general practitioners’ perspectives between current GP developmental pathway and ASP research pathway

The analysis yielded eight major themes and 23 subthemes across the two groups/pathways. Figure 1 illustrates the comparisons of barriers, enablers, and suggested improvements between the GPs allocated in the ASP and SaU pathways. Supplementary Table 3 provides a list of the themes and subthemes, with supporting quotes from participants shown in Supplementary Table 4.

### Theme 1.0 - overlapping enablers for both pathways

**Subtheme 1.1: Critical role of GPs.** There was recognition of the unique role of GPs in facilitating early identification and for intervening or providing the relevant care pathways. GPs stressed the importance for parents/caregivers to receive the right diagnosis and supports/services as early as possible for their children, especially those who have a “high likelihood” for developmental disabilities, such as autism. Participation of GPs in the ASP research was reported to improve their practice of regular monitoring/developmental surveillance regarding child developmental milestones, including early diagnosis and support/services.

“I think it’s very important, our role’s very important, because we need to start early, so that early intervention is good, because if it’s delayed, then time is lost. And then it’s harder to implement things when they’re older. So it’s always good to try and implement as early as possible”. (G45, Victoria)

**Subtheme 1.2: Enhanced communication between clinicians/health professionals.** It was considered important for GPs and other medical specialists to communication, collaborate/cooperate to deliver relevant healthcare services for their patients in the community. One of the enabling factors emphasised by GPs is prompt and reliable responses from specialists.

“The great advantage we have these days is that we communicate very quickly with specialists like paediatricians through social media. Many of the younger paediatricians are very quick to respond.” (G04, NSW)

**Subtheme 1.3: Mutual trust and relationship-building with patients.** GPs highlighted another important facilitator that creates positive experience/delivery of care for their patients, which involved a high level of trust between GPs and their patients. This established a quality patient-doctor relationship that in turn contributed towards an enhanced continuum of care.

“I think I’ve been looking after them for eight years, eight or nine years now. Three children have autism in their family. And so, I’ve seen them grow up. We’ve developed a trust with the children.” (G23, NSW)

**Subtheme 1.4: Having standardised screening tools.** GPs affirmed the benefits of using standardised screening tools as part of conducting child developmental checks. One GP stated that it guided him in initiating a conversation with the parents/caregivers:

“I usually take a bit of time there to ask the mum how they are going. And so I have a little chart on my wall, one of those standard developmental screening charts to remind me of what kind of milestones they should be achieving at each, at each level.” (G22, NSW)

### Theme 2.0 – ASP pathway-specific enablers

**Subtheme 2.1: Encouraging research towards further training and education.** Interest in supporting and participating in research contributed towards further training and education. It was noted that the study benefited and supported GPs in achieving their career goals, including the need to meet research work criteria and/or paediatric knowledge requirements as part of their ongoing GP fellowship training. The majority expressed a high interest being involved in relevant research studies.

“So, [for] the GP to be more involved, there needs to be more education and possibly need to start at a, you know, at a fellowship level. Before doctors are fully qualified. And look, maybe it’s already happening there, maybe with younger GPs but some sort of education around that. It wasn’t particularly well done when I went through, but that’s now a while ago, so whether it’s happening better now. But yeah, and awareness, I suppose.” (G36, Victoria)

**Subtheme 2.2: Specific assessment and resources.** Several GPs reported that they had limited knowledge about the specific characteristics of autism and other developmental disabilities in young children. However, participation in research such as this has allowed them to increase their understanding of some of the essential early autism developmental surveillance tools (e.g., SACS) and boost their confidence on identifying young patients who have a “high likelihood” of being autistic. Thus, they endorsed having the SACS Online assessment and resources as part of their additional evidenced-based screening tools.

“It was good training for me, because I got to pick up on a lot more characteristics of autistic kids that I wouldn’t have before.” (G34, NSW)

“It’s a reasonable tool. I just think it needs to be now sort out in a way that we can encourage more doctors to use again.” (G43, Victoria)

### Theme 3.0 - SaU pathway-specific enabler

**Subtheme 3.1: Importance of developmental screening.** Despite acknowledging their limited knowledge in paediatrics, many GPs said that they had been conducting routine developmental screening checks with their young patients as part of their regular practice. They emphasised the importance of ongoing monitoring for the parents/caregivers, since most have expressed concerns regarding whether or not their children are ‘on-track’ with their development.

“Usually will take place at all of the immunisations, most of the time. So that would be six weeks, six months, 12 months and 18 months, and then we’ll do another one around the three and a half to four year mark, that’s a standard. That is just literally questions to the parent, based on what should be there. The kid’s current developmental status, as usually followed in the blue book”. (G19, NSW)

### Theme 4.0 - overlapping barriers for both pathways

**Subtheme 4.1: Patient lack of understanding.** A major influencing factor on parental awareness of the ‘typical’ developmental milestones and support was the information provided to them by healthcare providers they encounter. Most GPs expressed concerns that the parents/caregivers they see have very limited understanding of developmental milestones, and yet they are expected to be aware of any developmental differences in their children, resulting in some emotional blame on GPs and placing them in a relatively uncomfortable position.

“I guess, if we put like blurbs about what’s the developmental stage of the kids at each age groups, like in a poster, and then the parents could read it while they’re in the waiting room, that might be helpful, basically, because, you know, like I said, if the parents have a two year old, [and a] six months [old], so it’d be great if I just put it there and while they’re in the waiting room waiting to be vaccinated. And that would be another thing.” (G06, NSW)

**Subtheme 4.2: COVID-19 lockdowns impacts.** One of the biggest challenges for GPs with study recruitment and follow up care was the lockdowns/restrictions imposed due to the COVID-19 pandemic. This prevented them from delivering care for their patients, as well as participating in the study.

“Because of COVID, all of their speech programs, all of them are closed. And they are not coming into GP practice as well, because they don’t want themselves or their children catch COVID as well. Definitely, a big barrier.” (G40, Victoria)

**Subtheme 4.3: Language and cultural barriers.** For NSW GPs, shared language and cultural barriers were identified as key factors for their routine consultation/practice with patients and participation in developmental screening programs. This involves communicating and directing CALD parents/caregivers about their child’s healthcare plan because they do not speak English. As one GP spoke about her general experience:

“Sometimes it’s hard to talk to them if they don’t speak the language. …. and you’re assessing them for speech. Because, you know, you’re asking them how many words are they saying? … And how do you accommodate that, because this one [*points to checklist*] shows up for Caucasian background where it’s just one language, one family, and we’re multicultural. So, the speech is a big deal.” (G06, NSW)

GPs also affirmed that parents/caregivers usually preferred simpler and straightforward information regarding their children’s health care plan. GPs often faced difficulties in delivering the right information to their patients due to limited awareness about child development or due to low health literacy. However, with relevant communication, it is possible to overcome this issue.

“The parents, the little bit uneducated one[s], they’ve found [it a] little bit hard to understand. But once [I] explained [to them], they were okay.” (G41, Victoria)

**Subtheme 4.4: Family financial and socioeconomic circumstances.** Other issues GPs alluded to were in relation to their patients’ (parents/caregivers) socioeconomic contexts, such as the location/distance of their service providers and their financial situations to afford a specialist appointment.

“…Some parents might have it better because they probably will get higher priority at [an OT Service] and stuff like that. But I’m just saying like a two person household where dad’s a meat-packer, and a mum who does just shift work at [a Supermarket], they will not be able to afford these things.” (G06, NSW)

Barriers like these often impede GPs to action their patient’s healthcare plan because they did not meet the criteria for payment of services under the Medicare Benefits Schedule (MBS) criteria (i.e., a list of health professional services that the Australian Government subsidises, such as consultations, diagnostic tests, and procedures/surgeries). Thus, they could not be directed to any other health assessments, unless these services can be covered by other public health funding, such as the National Disability Insurance Scheme (NDIS), a national government scheme that provides free support to eligible people with intellectual, physical, sensory, cognitive and psychosocial disability (Australian Government Department of Health, 2022) or paid for out-of-pocket by families.

“[The current] Medicare rebate for these cases is not sufficient. If they qualify for NDIS now, even with limited funding I think that helps a lot with access to treatment… Previously, dating back three or four years ago, a lot of the times they won’t seek treatment because of financial barriers.” (G05, NSW)

**Subtheme 4.5: Lack of workforce/resource.** Some GPs cited a lack of local specialist and allied health services as being a barrier for families seeking supports and services for their child.

“Parents have to travel half to one hour to go and see someone because they couldn’t get in near here.” (G35, Victoria)

GPs from both NSW and Victoria expressed concerns about long wait-times for specialist appointments due to a lack of workforce (i.e. paediatricians, psychologists, speech pathologists, etc.). This time delay creates another level of anxiety amongst families, as this may prolong the time from identification of a developmental concern to receiving the relevant assessments, diagnosis, and supports/services.

“If they’re waiting for public health or paediatrician, sorry. Well, yeah. Paediatrician, OT, whatever, speech pathologist. Yeah. By the time they get there, it’s been nine months.” (G27, NSW)

**Subtheme 4.6: Patient denial of (potential or actual) child’s diagnosis.** GPs also discussed some parents’/caregivers’ reluctance to accept that their child may have – or even only potentially have – a developmental disability. The intersectionality of families from CALD backgrounds was also brought up in this context.

“Parents, especially coming from other countries, they really don’t like me saying that their child, maybe is on the spectrum. And so they do try to deny and say maybe I’m thinking that because they are bilingual and because the child is shy.” (G40, Victoria)

**Subtheme 4.7: Complex navigation of the health system.** All GPs identified contextual challenges, such as organising referral forms, communicating with other healthcare professionals, and coordinating service availabilities for patients, due to the complexity in navigating the public healthcare system; especially when parents/caregivers need to access and utilise multiple services. One GP reflected on the fragmented process in NSW, which would often compound the burden of GPs as they would have to explain the entire patient flow/journey independently with each parent/caregiver they see, overwhelming families with a large amount of information to grasp.

“I can go forever with this. The GP is often pretty undervalued. Like it’s pretty easy, because you [are] meant to have this holistic approach to kids with developmental concerns or potential diagnosis, but it really happens like you’ve got this fragmentation of care. Like you have paediatrician says, go to the OT [occupational therapist] for that. OT will say “oh, that’s a speech problem, go to the speechie [speech pathologist]”. And then you often get parents who are a bit overwhelmed with the whole process. Like everyone deals with their specific thing, but no one often takes a step back and looks at everything.” (G23, NSW)

### Theme 5.0 - ASP pathway-specific barriers

**Subtheme 5.1: Lengthened screening time.** The ASP screening tools took longer for GPs to complete than they originally anticipated. This was compounded by the already brief consultation time-limit per patient. GPs’ time was frequently identified as a contextual barrier for GPs to conduct developmental checks with young children.

“And then when they come in, having the time to not only ask them the questions again, but having to go online,... So, it just all boils down to time. And sometimes I find that because we’re so busy, I don’t actually have time to fill out that second part of the survey.” (G24, NSW

**Subtheme 5.2: Technical implementation issues.** Some GPs from NSW experienced *technical* problems when receiving their patients’ screening results. Although this was rectified early in the study, the flow of digital screening questionnaires was impacted for some GPs early on.

“It was a bit clunky at the beginning that it was hard to you know, they’re meant to fill out the questionnaire and then we get an email back straight away with the result, but sometimes they didn’t come back.” (G22, NSW)

### Theme 6.0 – overlapping recommendations for both pathways

**Subtheme 6.1: Need to clarify the role of GPs.** Several GPs reported that while they often find themselves in a ‘co-ordinator’ role for families whose children have developmental concerns, they do not have the time, funding, nor sufficient knowledge of the process from identification to diagnosis to supports/services to fully execute this role.

“I don’t think GPs can help much at the moment unless I see it in a different form, NDIS early developmental intervention. We don’t have the expertise or the time, or probably … definitely don’t have the funding, so I don’t think you’re going to get any motivation to see GPs in this sphere.” (G44, Victoria)

**Subtheme 6.2: Need for further training and education.** Most GPs are not well-trained in paediatrics. Universal developmental surveillance/monitoring would usually require further knowledge and training about developmental milestones. Our GP cohort stressed the importance of up-to-date, additional education and relevant training.

“I think, I would just have to admit that I lack the skills, I feel I lack the skills. I couldn’t tell a parent how to improve speech or, you know, it’s just not something that I’m trained in and have any exposure to, really… I would pretty much refer and support, but to specifically provide some instruction on, I’d hesitate, because I haven’t had the training … with the unacceptable delays in time, and crucial time that, you know, we could be doing something, if we were upskilled and we’re able to assist and put people in the right direction of what they can do while they’re waiting. Absolutely.” (G34, Victoria)

**Subtheme 6.3: Need for a comprehensive, streamlined process.** The need for an explicit, streamlined process to deliver developmental surveillance in the general practice setting was recommended by GPs, to limit the currently convoluted healthcare system and reduce their already high workload.

“Well, identifying and putting them into the right streamline are very important things that should happen to a child which is best not to fall under my responsibility, but I’m the facilitator, to some extent, of referrals.” (G27, NSW)

**Subtheme 6.4: Need for funding.** Some GPs acknowledged the need for additional funding for measures in the primary healthcare model, to address the contextual barriers faced by parents/caregivers. For example, as one GP noted, the addition of an MBS item for providing more than just a ‘once-off’ health assessment in the context of a broader knowledge of the child’s development.

“So, if you had provision to have a Medicare item number for say a health assessment-type thing in which you could do for a developmental assessment that would make a massive difference, because you [would] train your nurses up. And yeah, [it] just makes it more feasible in [the] general practice model.” (G36, Victoria)

**Subtheme 6.5: Need for digital developmental screening.** Most GPs agreed that developmental surveillance tools can be completed electronically by parents/caregivers while waiting for their GP appointments. This would allow more time for discussion between families and the GP regarding the child’s healthcare needs and maximising their consultation time.

“I think the computer and everything online is great now because people can actually visualise it in front of them. So, I actually use the computer and online services quite a lot during my consultations… So, in terms of developmental screening, it’s, I find it really useful when there’s … I show parents, they, you know, there’s a table with the age and then you know, the milestones and that sort of stuff.” (G24, NSW)

### Theme 7.0 - ASP pathway-specific recommendation

**Subtheme 7.1: In-clinic administration support.** To implement a streamlined and consistent ASP protocol, a few NSW GP highlighted the need to have additional staff and resources to assist with the administrative processes and coordinate the program with GPs and staff members.

“If we had someone with a, with an iPad who could actually go and screen in the waiting room, that might be an option utilising these opportunities during those immunisation periods to screen those patients when they come in, it could. Also having sort of like a navigator person in the waiting room, absolutely. Anything to help take the burden off the doctors and the nurses would be ideal.” (G32, NSW)

### Theme 8.0 - SaU pathway-specific recommendation

**Subtheme 8.1: Need for a quick-reference parental/caregiver information.** One barrier identified was the need for simplified and clearer information tailored for parents/caregivers about universal developmental milestones. This subtheme was recommended by GPs to be implemented in the public or community healthcare systems in order to increase parental/caregiver awareness, knowledge, and confidence about monitoring their children’s growth and development.

“If it’s a parental anxiety, some of the parenting resources can be useful, just so they can get an appreciation of what sort of ‘normal’ development is and what the timeframe is. And I think that’s more of an issue for parents where it might be their first child, and it’s like, well, you know, are things on track, is it something we need to be worried about and, you know, sometimes getting another source other than me to confirm what I’m saying can be sort of reassuring in that sort of context.” (G33, Victoria)

## Discussion

A number of factors emerged regarding GPs’ perceptions and experiences about feasibility after they had participated and implemented the program’s developmental surveillance assessments with families of children potentially on the autism spectrum. The findings from this study highlighted a number of enablers, barriers, and improvements in both the digital universal developmental surveillance and the routine care pathways. Although GPs in both groups identified overlapping barriers and facilitators, GPs encountered greater challenges than enablers in both the current and ASP settings while also providing their encouragements/suggestions for improvement to the overall surveillance program.

Indeed, the themes and subthemes reported in this paper are similar and complimentary to the findings that emerged from our parent/caregiver cohort [17]. For instance, GPs affirmed that the COVID-19-associated factors have impacted their practice and this was affirmed by all the parents/caregivers who participated in this surveillance program. Other significant factors that require consideration in future implementation include addressing the complexity of the health system and navigation support, technical issues, lengthening times of child developmental consultation, resources and funding alongside the need for educational resources/guides and guidelines.

GPs tended reported that the quality of their healthcare delivery towards patients is grounded in mutual trust and positive relationships, thereby improving the patient’s overall healthcare experience and wellbeing. In this regard, previous studies have shown the critical role of trust in the doctor-patient relationship through open communication, engagement, and shared decision-making [29, 30]. Furthermore, GPs felt encouraged and enthused about their research participation as it not only developed their clinical and patient engagement skills regarding promoting early diagnosis, supports and services for children found to have a likelihood of being autistic, but it also provided them an opportunity to learn more about the field, contributing towards their further education and training in paediatrics.

Implementing a digital developmental surveillance system within the primary healthcare setting is another innovative pathway for GPs to engage with and deliver care for their patients, in a systematic manner using standardised tools. One such program developed by our team, the ‘Watch Me Grow-Electronic’ platform (WMG-E), empowers parents/caregivers to engage in developmental surveillance using opportunistic contacts such as vaccination visits to complete developmental checks digitally and for this to be done in the family home or in the community [31, 32]. Once engaged, the system automatically sends reminders to re-take the developmental checks. This provides the opportunity for ongoing developmental monitoring at the recommended ages and stages (6-, 12-, 18-, 24-, 36-, 48-, and 60-months). This is similar to the Victorian Maternal and Child Health service, where all Victorian children are monitored at 10 ‘key age and stage’ (KAS) assessments (after birth home visit, 2-week, 4-week, 8-week, 4-month, 8-month, 12-month, 18-month, 2-year, and 3½-year home visits) [33]. KAS assessments cover child development and child and parental/caregiver health and wellbeing, as well as the SACS assessments at 12-, 18-, and 24-months, [34–36] which GPs were trained on and implemented in the current study.

Several electronic or web-based screening tools have been used in various healthcare settings, including mental health services and hospital/ambulatory care settings [37–39]. However, technical issues must be rectified before digital implementation of such monitoring tools can be introduced and utilised by patients within GP waiting rooms. The need for an in-depth, technical pilot and/or trial is therefore warranted.

Despite facilitating factors and positive experiences, GPs also highlighted barriers including the language and cultural beliefs about developmental screening, socioeconomic situations of the families, lack of availability of health specialists’, and the complexity of the health system. These findings were echoed by a similar, qualitative study conducted in NSW which also highlighted the practical challenges such as limited knowledge and uptake of the use of the recommended screening tools as part of child developmental surveillance by service providers [16]. Access issues, including transport and other perceived barriers for parents/caregivers to access child developmental checks/services, parental/caregiver choices and engagement in developmental screening, and parents’/caregivers’ knowledge, understanding, and beliefs of the need for screening were also noted in the previous study [15].

Another key barrier GPs encountered was in relation to the COVID-19 pandemic lockdowns/restrictions, which affected routine patient care. Changes in practice management and in consultation strategies included a major switch towards telephone triage/consultations and/or patient care occurring in carparks; however, acute/chronic care delivery was mostly postponed and recruitment of patients to the study was decreased substantially during the pandemic. Other primary health research studies confirmed these same issues [40, 41]. COVID-19 lockdowns have had a profound impact on the core competencies of primary care, with GPs primarily concerned about the continuity of regular care and the consequences of these on patient outcomes and wellbeing. These may become a threat for the general health of the population and for the provision of primary healthcare in the near and distant future.

General practice is a relatively busy space, with GPs often having to manage multiple processes and tasks to get routine patient care completed [42, 43]. The notion of having an in-clinic administrative liaison personnel was suggested by GPs from NSW to implement a digital solution protocol in GP waiting rooms which would reduce the workload of GPs/clinicians in the primary healthcare setting. This was highlighted by Neuwelt et al. [43] reinforcing the critical role of practice receptionists as the first step in the patient care pathway, bridging healthcare system and the community. For general practice to be patient-centred and improve accessibility for the most vulnerable, the support of clinical receptionists was considered essential.

Additionally, this study identified several health system barriers, such as long waiting times, service fragmentation and lack of specialist resources, particularly in the SaU pathway. These barriers have also been reported in recent studies about clinician perspectives about the care of children and adolescents with mental health conditions [44, 45]. The consistency of these findings across the different studies suggests that these challenges should be considered a priority in future planning for services designed to help parents/caregivers and their children. The perceptions of GPs in the current study highlight the extent to which they would like better access and optimal care for their patients, especially by implementing an easy-to-access, coordinated, specialised, and effective pathway/support to specialist consultation (e.g., paediatricians and allied health professionals).

The GPs in our study observed that parental/caregiver understanding of child developmental milestones is an important factor contributing to a smooth, streamlined pathway of developmental surveillance and better patient care. According to the Australian Commission on Safety and Quality in Health Care, [46] the health literacy environment within the healthcare context either makes it easier or more challenging for consumers to understand health information, to make effective decisions and take appropriate action about their healthcare. Indeed, studies have shown that lower levels of health literacy amongst patients are associated with lower levels of knowledge of care and poorer health outcomes [45, 47, 48]. Hence, the need for simpler and straightforward information guides for parents/caregivers must be considered to increase knowledge and to enable better informed decision-making skills around child development. This will allow GPs and parents/caregivers to better collaborate on shared decision making and to deliver positive healthcare experiences and child developmental outcomes.

In the current study, GPs also emphasised the need for further training and education around child development. As alluded by Price and Rechert, [49] continuous education and training is an absolute necessity for any healthcare professional who wants to provide high-quality patient care, since healthcare providers must regularly keep up with new skills, techniques, and technologies.

### Implications for health practice and policy

The findings from this study indicate the need for increased awareness about the importance of developmental screening/surveillance, including for autism, amongst primary care clinicians such as GPs. This should focus on providing education and training about developmental milestones in general and early signs of autism to facilitate early identification alongside pathways for effective support and services for those children identified to have developmental difference or found to have a likelihood of being diagnosed as autistic. Unifying the developmental surveillance approaches using relevant health policies and guidelines (e.g. National Guideline for the assessment and diagnosis of Autism Spectrum Disorders in Australia) [50] can help increase the capacity of primary healthcare professionals to support early identification of developmental disabilities, including autism. However, it is to be noted that time is a critical barrier as most GPs in this study felt ‘insufficient time’ within a typical appointment as a significant issue. Hence countries looking to improve early and accurate identification of developmental disabilities will need to put in place appropriate national policies to provide resources and remuneration for clinicians and consistent use of developmental screening/surveillance tools during opportunistic contacts such as immunisation visits.

It is also important to increase awareness among parents/caregivers and families, including those from CALD communities, by making available freely-accessible screening tools (such as the ASDetect mobile application, which is based on the SACS tool), [51] and resources on the importance of early developmental monitoring and milestones. In this context we would like to highlight that while this paper has focussed on the feasibility of carrying out developmental checks using opportunistic contacts by the GPs, the same can also be applied to alternate opportunities that are available through nurses and community-based childcare and other service providers who come in contact with very young children. Several countries and jurisdictions rely on other professionals, including nurses and community health workers, to undertake early screening and interventions. However, in Australia since GPs form the only ‘universal’ health service system available to all children and families (regardless of their cultural, linguistic, socioeconomic or geographic background), our study on investigating the feasibility of systematically doing developmental checks using opportunistic contacts within the GP setting has implications for equitable access to early identification of developmental differences across Australia, and also internationally. Further, the findings highlight the critical need to increase the number and capacity of professionals (GPs, nurses, allied health, early childhood education/childcare staff) for culturally sensitive and linguistically congruent assessments in order to enable equitable access to early identification of developmental needs for all children.

Some GP clinics that have participated in our project are very much still comfortable in paper-based work and were not comfortable switching to online mode of operation. The shift to digital delivery means greater efficiencies, specifically in terms of time and instant communication of results and recommendations with the relevant healthcare professionals. However, the pandemic has seen a shift in the general attitude towards digital systems with telehealth practice becoming increasingly available and acceptable to both patients and to service providers [52]. Hence it is expected that more GPs if not all will be incentivised to transfer their clinical and administrative operations via technical/digital programs or applications as part of the post-pandemic re-set of service delivery.

### Strengths and limitations

There are a number of strengths to this study, including the use of a semi-structured interview guide for a relatively high number of participating GPs. Further, we maintained a high level of interpretive rigour and trustworthiness and quality of the study through an awareness of reflexivity, data auditing and use of quality control measures as well as coding of the analysis by a researcher independent of the data collection.

There are also some limitations that need highlighting. One of the limitations is that the GPs who participated in this qualitative study were willing/interested and there may be geographic differences based on state processes and also remoteness and other characteristics. However, this study was aimed to ascertain the feasibility and also potential enablers and barriers to implementation as pilot work before scaling up the program to other contexts and settings. Further, since the GPs were from two arms of a wider cRCT program, we are confident that the responses are generalisable to the contexts in which this study was conducted and the leanings from this pilot work could still inform dissemination of the program to other contexts and settings. For example, some of the findings observed in this study regarding barriers and enablers may be related to local issues at the study sites in either NSW (South-West Sydney) and/or Victoria (metropolitan Melbourne) only, other issues relating to the process, technology and funding are relevant to other parts of Australia and even globally. Further, the findings are consistent with previous research in Australia and internationally about the use of developmental and autism-specific screening tools [15–17, 53, 54]. Also, the unusual circumstances over the study period caused by the COVID-19 pandemic deserve mention. Despite significant project planning and risk mitigation measures put in place by the study team, the severe and ongoing impacts of the COVID-19 pandemic on both general practices and families had a significant detrimental impact on the completion of the main cRCT study. Given the challenges experienced by the community, and in particular families with young children during the extensive lockdowns/restrictions, recruitment was significantly impacted as families with young children avoided attending GP clinics. Hence, there were clinics that could not commence the study or recruit any participants, which may have led to a skewed sample in terms of GPs’ responses towards the research study setup. Notwithstanding these limitations, the study was able to elicit valuable information from GPs about the facilitators, barriers, and recommendations for improvements to the implementation of digital developmental surveillance programs in the primary care setting.

## Conclusion

The findings from this qualitative study provides valuable insights into the perceptions and experiences of Australian GPs regarding the contextual enablers and barriers that impact their participation in developmental surveillance programs, and in particular, the potential and beneficial usage of digital screening tools for developmental surveillance including the early identification of autism.

Additional policies and systems-based research are needed, particularly in relation to the role of not only GPs but other community based services that interact with preschool children and their families. We believe there needs to be a multi-prong approach of using every opportunistic contact in the preschool period for identifying developmental differences as they emerge. In this regard, the team is also trialling developmental checks using the Watch Me Grow web link in other settings using services that the families are already engaged with and trust. These include multicultural playgroups, early childhood education centres and NGOs providing social care and other community-based services to empower and engage families in developmental monitoring of their children [31]. Further, the model of using opportunistic contact with very young children during immunisation and other mandatory or highly-utilised service interactions for engaging families in developmental checks will be of interest to the global audience of primary care practitioners regardless of the context or other differences within which they operate. Since immunisation has an uptake of > 90% in most countries, including in low and middle income countries, this model of dove tailing developmental checks during immunisation could offer a unique opportunity to reach most children universally, including those who are typically hard to reach.

There is an urgent need to improve GP’s skills and confidence in the early and accurate identification of developmental disabilities, including autism, and their knowledge of pathways for providing care for these children. To undertake developmental surveillance and the early identification of autism, GPs require updated training on the early signs of developmental disabilities as well as how to raise developmental difference with parents/caregivers. Further, culturally sensitive ways of supporting children and their families is important, along with access to accurate evidence-based early identification tools. As time and financial constraints emerged as barriers to the implementation of developmental monitoring, addressing this through the creation of remuneration for GPs to specifically undertake child developmental surveillance will facilitate access to early identification of autism. Furthermore, integrated referral pathways and models of care are also critical to address some of the identified barriers. Overall, this qualitative study is one component of a larger study that, together with the other components, is expected to provide much needed evidence on the implementation of an effective national, digital developmental surveillance program for early identification of developmental disabilities, including autism, in the primary care setting. Thus, the model being tested here has the unique potential to systematically reach all children/families by using GPs or the equivalent primary care service providers that are universally available in a given population. Further the utilisation of universal service providers in the community who engage with very young children and their families for immunisation or other routine service contacts will provide a feasible framework for equitable access to early developmental checks and a systematic opportunity for early identification of all developmental differences, such as autism, early in life.

### Electronic supplementary material

Below is the link to the electronic supplementary material.

**Supplementary Material 1: Supplementary Table 1. Full interview guide and prompts for GPs.**

**Supplementary Material 2: Supplementary Table 2. Standards for Reporting Qualitative Research Checklist.**

**Supplementary Material 3: Supplementary Table 3. Overview of themes and subthemes.**

**Supplementary Material 4: Supplementary Table 4. Themes, subthemes and reference quotes of participating general practitioners’ perspectives and experiences.**

## Data Availability

All data generated or analysed during this study are included in this published article and its supplementary information files.

## Declarations

### Provenance and peer review

Not commissioned; externally peer reviewed.

## Abbreviations

ASP: Autism Surveillance Pathway
ASQ: Ages and Stages Questionnaire
ASQ-SE: Ages and Stages Questionnaire-Social and Emotional Scale
Autism CRC: Cooperative Research Centre for Living with Autism
CALD: Culturally and Linguistically Diverse
COVID-19: Coronavirus Disease 2019
cRCT: cluster Randomised Controlled Trial
GP: General Practitioner
KAS: Key Age and Stage
LTSAE: Learn The Signs. Act Early
MBS: Medicare Benefits Schedule
NDIS: National Disability Insurance Scheme
NSW: New South Wales
PEDS: Parents’ Evaluation of Developmental Status
Q-CHAT-10: Quantitative Checklist for Autism in Toddlers
SACS: Social Attention and Communication Surveillance
SAU: Surveillance as Usual
WMG: Watch Me Grow
WMG-E: Watch Me Grow-Electronic
