# Supplementary material for: General practitioners’ perspectives regarding early developmental surveillance for autism within the australian primary healthcare setting: a qualitative study
Source: BMC Prim Care. 2023 Aug 10;24:159. doi: 10.1186/s12875-023-02121-6 (PMC10416397; doi:10.1186/s12875-023-02121-6)
Supplement: Supplementary file 1 — Supplementary Material 1: Supplementary Table 1. Full interview guide and prompts for GPs. [file 12875_2023_2121_MOESM1_ESM.docx]

**Supplementary Table 1.** Full interview guide and prompts for GPs.

| Main questions for GPs | Prompt or follow-up questions |
| --- | --- |
| Describe your experience of conducting childhood developmental screening/surveillance (DS) in your practice. | - Can you give me an example or some examples when you have screened the child and tell me how that worked out? - When do you conduct this screening? - When are you more likely to conduct screening? - Is this something you do routinely, if not why? - How do you conduct this screening? E.g., what tools do you use, how much time, other factors, etc. - How do you work with the Practice Nurse (PN) or other staff at your practice when conducting DS? - How do you work with other health practitioners such as Child and Family Health Nurses (CFHNs), speech pathologists, Paediatricians when conducting DS and how accessible are they? - How do you see your role in conducting DS? |
| Describe what other factors may assist you to conduct DS in your practice? | - Is there anything practical or structural support that you require to conduct DS in your current practice? - Can you suggest what is needed to help GPs/PNs to implement/adopt DS routinely in general practice? |
| What barriers did the COVID pandemic and associated changes pose on conducting DS at your practice?  Were there any specific enablers that you found helpful in conducting DS? | - Can you give me an example of what made it difficult to conduct DS that was specifically linked to the pandemic? - How about any changes linked to the pandemic that made it easier for you to conduct DS (e.g. access to Medicare billing for telehealth)? |
| Describe your experience in managing children whom you (or their parents) identified as having a specific developmental concern. | - Can you give me an example and describe how you went with this child and who was involved? - Do you conduct further assessment in your current practice? - Do you conduct this assessment yourself and if not why? - And if you refer, to whom or what service? - How do you work with other staff (e.g., PNs) within your practice or with other health professionals outside your practice in these situations? |
| How do you perceive your role in Early Intervention (EI) for child developmental issues? *FYI The National Disability Insurance Scheme (NDIS) has made available early childhood early intervention (ECEI) services for children aged under seven years of age with a developmental delay or disability.* | - Is there anything practical or structural that you require to implement EI in your current practice? - Can you suggest what is needed to help GPs or PNs to implement/adopt EI routinely in general practice? |
| Overall, can you describe your role in providing ongoing care for a child with developmental disability (CWDD) within your practice? In particular, we are interested in how you see the extent of your involvement after the children are referred to other services e.g. specialist paediatric or others. | - What is currently your experience with CWDD who attend these services? Can you please give an example of this? - Is there anything practical or structural within your practice currently that can assist you in providing child developmental surveillance and ongoing care for children with developmental concerns? - How would you like to be involved in the care of children with developmental conditions such as autism? - Can you suggest what is needed to help GPs (and PNs) to play an ongoing role in the care of children with developmental conditions in general practice? |
| Additional questions for GPs in the ASP pathway to obtain views on their experiences with the study tools and procedures. | |
| Can you describe your experience of participating in the ASP subgroup of this study? | N/A |
| Were there any issues; what suggestions do you have to address this to resolve this? | N/A |
| What were your experiences like with completing the SACS Online assessment with children? | - Were there any particular barriers or enablers for completing the SACS Online assessment? |
| What were your experiences like with the parent/caregiver questionnaires – the Q-CHAT-10, ‘Learn the Signs. Act Early’, and the PEDS? | - Did you notice any positive or negatives for parents in completing these questionnaires? - Were there any particular barriers or enablers for *yourself* relating to these tools? |
| What are your thoughts on using these clinician and parent-completed tools in the future for conducting childhood developmental screening? | N/A |
| Anything else to add? | N/A |
