# Supplementary material for: General practitioners’ perspectives regarding early developmental surveillance for autism within the australian primary healthcare setting: a qualitative study
Source: BMC Prim Care. 2023 Aug 10;24:159. doi: 10.1186/s12875-023-02121-6 (PMC10416397; doi:10.1186/s12875-023-02121-6)
Supplement: Supplementary file 2 — Supplementary Material 2: Supplementary Table 2. Standards for Reporting Qualitative Research Checklist. [file 12875_2023_2121_MOESM2_ESM.docx]

**Supplementary Table 2.** Standards for Reporting Qualitative Research Checklist

| **Index** | **Topic** | **Section(s)** | **Page No. in the main manuscript file** |
| --- | --- | --- | --- |
|  | Title and Abstract |  |  |
| 1 | Title | Title Page | 1 |
| 2 | Abstract | Abstract | 3 |
|  | Introduction |  |  |
| 3 | Problem formulation | Background | 5-7 |
| 4 | Purpose or research question | Background – Research Aim | 6-7 |
|  | Methods |  |  |
| 5 | Qualitative approach and research paradigm | Methods | 7-10 |
| 6 | Researcher characteristics and reflexivity | Methods – Data Analysis | 9-10 |
| 7 | Context | Methods – Research Context | 7-8 |
| 8 | Sampling strategy | Methods – Participant Recruitment and Interviews | 8-9 |
| 9 | Ethical issues pertaining to human subjects | Ethics Approval and Consent to Participate | 31 |
| 10 | Data collection methods | Methods – Participant Recruitment and Interviews | 8-9 |
| 11 | Data collection instruments and technologies | Methods, Table 2, plus Supplementary Table 1 | 7-10 |
| 12 | Units of study | Methods – Participant Recruitment and Interviews | 8-9 |
| 13 | Data processing | Methods – Data Analysis | 9-10 |
| 14 | Data analysis | Methods – Data Analysis | 9-10 |
| 15 | Techniques to enhance trustworthiness | Methods – Data Analysis | 9-10 |
|  | Results |  |  |
| 16 | Synthesis and interpretation | Results, Figure 1, Supplementary Table 3 | 10-21 |
| 17 | Links to empirical data | Results, plus Supplementary Table 3 | 10-21 |
|  | Discussion |  |  |
| 18 | Integration with prior work, implications, transferability, and contribution(s) to the field | Discussion | 21-28 |
| 19 | Limitations | Discussion – Strengths and Limitations | 27-28 |
| 20 | Conclusion | Conclusion | 28-30 |
|  | Other |  |  |
| 21 | Conflicts of interest | Competing Interests | 31 |
| 22 | Funding | Funding | 31 |

**Reference:** O’Brien BC, Harris IB, Beckman TJ, *et al.* Standards for reporting qualitative research: a synthesis of recommendations. *Acad Med.* 2014;89:1245–51.
