# Supplementary material for: General practitioners’ perspectives regarding early developmental surveillance for autism within the australian primary healthcare setting: a qualitative study
Source: BMC Prim Care. 2023 Aug 10;24:159. doi: 10.1186/s12875-023-02121-6 (PMC10416397; doi:10.1186/s12875-023-02121-6)
Supplement: Supplementary file 3 — Supplementary Material 3: Supplementary Table 3. Overview of themes and subthemes. [file 12875_2023_2121_MOESM3_ESM.docx]

**Supplementary Table 3.** Overview of themes and subthemes.

| **Code** | **Theme** |  | **Subtheme** | |
| --- | --- | --- | --- | --- |
|  | | | ASP research pathway | SaU pathway |
| **1.0** | **Overlapping enablers for both pathways** | | | |
| 1.1 |  |  | Critical role of GPs | |
| 1.2 |  |  | Enhanced communication between clinicians/health professionals | |
| 1.3 |  |  | Mutual trust and relationship-building with patients | |
| 1.4 |  |  | Having standardised screening tools | |
| **2.0** | **ASP pathway-specific enablers** | | | |
| 2.1 |  |  | Encouraging research towards further training and education |  |
| 2.2 |  |  | Specific assessment and resources |  |
| **3.0** | **SaU pathway-specific enabler** | | | |
| 3.1 |  |  |  | Importance of developmental screening |
| **4.0** | **Overlapping barriers for both pathways** | | | |
| 4.1 |  |  | Patient lack of understanding | |
| 4.2 |  |  | COVID-19 lockdowns impacts | |
| 4.3 |  |  | Language and cultural barriers | |
| 4.4 |  |  | Family financial and socioeconomic circumstances | |
| 4.5 |  |  | Lack of workforce/resource | |
| 4.6 |  |  | Patient denial of (potential or actual) child’s diagnosis | |
| 4.7 |  |  | Complex navigation of the health system | |
| **5.0** | **ASP pathway-specific barriers** | | | |
| 5.1 |  |  | Lengthened screening time |  |
| 5.2 |  |  | Technical implementation issues |  |
| **6.0** | **Overlapping recommendations for both pathways** | | | |
| 6.1 |  |  | Need to clarify the role of GPs | |
| 6.2 |  |  | Need for further training and education | |
| 6.3 |  |  | Need for a comprehensive, streamlined process | |
| 6.4 |  |  | Need for funding | |
| 6.5 |  |  | Need for digital developmental screening | |
| **7.0** | **ASP pathway-specific recommendation** | | | |
| 7.1 |  |  | In-clinic administration support |  |
| **8.0** | **SaU pathway-specific recommendation** | | | |
| 8.1 |  |  |  | Need for a quick-reference parental/caregiver information |
