# Supplementary material for: General practitioners’ perspectives regarding early developmental surveillance for autism within the australian primary healthcare setting: a qualitative study
Source: BMC Prim Care. 2023 Aug 10;24:159. doi: 10.1186/s12875-023-02121-6 (PMC10416397; doi:10.1186/s12875-023-02121-6)
Supplement: Supplementary file 4 — Supplementary Material 4: Supplementary Table 4. Themes, subthemes and reference quotes of participating general practitioners’ perspectives and experiences. [file 12875_2023_2121_MOESM4_ESM.docx]

**Supplementary Table 4.** Themes, subthemes and reference quotes of participating general practitioners’ perspectives and experiences.

| **Code** | **Theme** | **Subtheme** | **Participant responses (participant ID number, state)** | |
| --- | --- | --- | --- | --- |
|  | | | ASP research pathway | SaU pathway |
| **1.0** | **Overlapping enablers for both pathways** | | | |
| 1.1 |  | Critical role of GPs | Our role is to be able to be screeners in the community. And we are also information providers, we are points of reference for hearing parents who are concerned or teachers. So, I think that we’re central to the actual general practice usually. (G37, Victoria)  I also like your program. The child got huge benefit. This is why that I'm saying, how much the intervention earlier, the more benefits. He started everything, was so quicker for him. He got his NDIS so quick, and everything was very good. And I think this will also benefit GPs (G40, Victoria) | |
| 1.2 |  | Enhanced communication between clinicians/health professionals | The great advantage we have these days is that we communicate very quickly. With specialists like paediatricians through social media. Many of the younger paediatricians are very quick to respond. So, I can actually put in a brief report about the patient“… "I have child with...a four-year-old who is not doing such and such. What do you think?” And they're very quick to provide advice, which are usually within a few hours. (G04, NSW)  I think around our area is fine. Allied health/specialists quite accessible, no matter your demographic. (G44, Victoria) | |
| 1.3 |  | Mutual trust and relationship-building with patients | Parents that who are coming and seeing me during the pregnancy and after birth, and I am raising my concern, they are very welcome. I found that they've more patience because it looks like that they really have a more trust, rather than then wanting for the second opinion, either way or not. (G40,  Victoria)  Luckily, most of us have great relationships with our patients. So, we get to encourage them to be a key part of that process. And touch base regularly. Yeah, that's important, it’s … I mean, we're blessed with that. (G43, Victoria)  I think as GPs we are pretty privileged that we get to grasp a broader understanding of family dynamics. Like you might see the other people in the family, you know, that mum or dad might have their own issues and that's why they can come and access as much therapy and intervention. (G23, NSW) | |
| 1.4 |  | Having standardised screening tools | I think I would love for you all to sort of have these programs on a regular basis, so that we could utilise these programs and the ability to get quickly in and, thereby, sort of implement the strategies early, having a structure like what your setup is very good. And I think it will be pretty useful for all general practice. (G45, Victoria)  So, in general, because we do that as part of a standard check-up anyway, so each time a child comes in for these checks we just ask a set of general questions in terms of their development. So, their motor skills, their social skills, their language and speech development, that sort of stuff. So, I think in terms of this study, it fits in perfectly with this study anyway. So, there's, there's nothing extra that we have to do except for the survey that they have to answer outside. (G24, NSW) | |
| **2.0** | **ASP pathway-specific enablers** | | | |
| 2.1 |  | Encouraging research towards further training and education | So, when I have seen you do [the] research project, I really felt that it's uh very good that somebody is thinking to train and understand how to pick up developmental issues by the age of two [years], so it is a very, very important thing. So, in medical school, I mean postgraduate GP training, of course in paediatrics training that will do all the training but in GP training, it needs to be included how to pick up, early pick up of [a] developmental issue. (G41, Victoria)  I'm a very strong advocate in education for our medical students and also for GPs’ need to pass the fellowship exams. So, what I'd like, if it could be put in a syllabus about the RACGP syllabus for paediatrics, a standard approach to looking at milestones and giving us a little bit more education, I've trained as a GP back in the nineties, we didn't have that. Now we have the diploma of paediatrics, but not all doctors are going to do it. I don't think I need to do it, but it'd be lovely if we could have updates on paediatrics milestones and maybe then put it in a way for young doctors coming through as part of the syllabus, paediatric milestones. I think that would be great, you know, if the research was done and then that way, we could then have a standard thing and then it will prompt me as a supervisor to make sure that my registrar is competent in those things. (G26, NSW) |  |
| 2.2 |  | Specific assessment and resources | I think it's been a really good eye opener of things to look out for. I think you're going to find that it actually ends up being the criteria you're looking at, I think [this tool] is going to be very helpful for people. And I think it's something that I'm more aware of, and will be keeping in the back of my mind when I'm seeing young kids, as a way of having a better understanding [of] who's at risk, because I do think that you are going to find you get good results from this when you get your results in. … as I say, just that it's made, improve my awareness and hopefully my ability to pick things up a bit earlier as well. (G35, Victoria)  When I had your software package, I would use the prompts that you have, which will allow us to specify and that will also allow me to get some clarity on this situation… The tools were excellent. (G37, Victoria)  I found that I keep emphasising the resource. I just found that so useful. Sure, and I think, you know, you can read about things. But those short videos were so helpful to really see what it is we're looking for, as I say, you can read about things, but it's just not the same as seeing something, very well done, very concise, easy to access, and I think all GP should have access to that sort of learning tool. (GP34, Victoria) |  |
| **3.0** | **SaU pathway-specific enabler** | | | |
| 3.1 |  | Importance of developmental screening |  | So in general I think when the children come for their routine check-ups, so the six week check-up, and then they come for the four months, six months, 12 months, 18 months. So I usually take a bit of time there to ask the mum how they are going. And so I have a little chart on my wall, one of those standard developmental screening charts to remind me of what kind of milestones they should be achieving at each at each level. (G22, NSW)  We try and do a quick developmental screening and issues of concern. Well, we particularly do it when it's a 12 month appointment. And again, at eighteen months, when they come in for the 18 month  immunisations or when they attend the GP clinic, not the immunisation clinic, most of us then also re-evaluate them as to whether there are any suspicious features or specific concerns (G43, Victoria)  So usually from … I do several of the screens. One is on the six weeks old, one is on the four month old, one is on the six month, one is on the twelve [months], one is on the 18 [months], one is on the 2 [years]. So on each of them, I usually check the milestone in all comprehensive, not only on the physical, also on the mental. So check for the … if they have the proper eye contact, about the language, about the comprehensive spectrum that I have as a chart. So to check if they are both developmental, mental developmental, milestone is okay. And physical milestone is okay, based on their age. (G40, Victoria) |
| **4.0** | **Overlapping barriers for both pathways** | | | |
| 4.1 |  | Patient lack of understanding | And the parents, many parents will attribute one end of the spectrum or the other, they live their life to believe that their kid’s absolutely normal and perfect, or they're overly worried and overly anxious, and overly analysing anything as a sign of something. And for both ends of that spectrum, slightly different approaches, and concerns. But teaching skills, social communication and social interactions and teaching strategies on behaviour is definitely worthwhile.(G43, Victoria) | |
| 4.2 |  | COVID-19 lockdowns impacts | Everything about COVID it has, it's more difficult. It's more time consuming. Even the phone calls by the time that you actually get them online. It's not always a convenient time ... I'm here sometimes till 7, 8, 9 pm doing the phone calls. Then there is the need to do whatever it is they need to do. I get it to, will you pick it up? Am I mailing emailing, faxing? It's actually been more time consuming than when they were here and they left right. With everything being done that was required to be done. (G27, NSW)  So for about five, five or six months there, we, we couldn't conduct a proper exam, you know, even on our regular patients, because they were scared to come in. Even immunisations were postponed. Even regular immunisations were being postponed at that time. And it was locked down. And so most of it is phone. And, you know, there's only so much you can do on the phone. Yeah. So I think COVID would have substantially delayed a lot of a lot of those prep years kids, you know, they were at home. Yeah, pretty much all of last year. So it's really now things would be identified. (G42, Victoria)  I did have trouble getting them to get their consent forms looked at and signed up. Because partly with the whole COVID lockdown, the clinic wasn't running as it normally is, people sitting in the waiting room, they only come in the minute you've starting your consult, and so on. So that didn't help. (G35, Victoria) | |
| 4.3 |  | Language and cultural barriers | Caretaking facilities would be helpful sometimes. In this area it's white, it's basically everybody speaks English. I've got maybe, no, I don't know of any young families where the patient, the parents don't speak good English. Even the older ones, the oldies, usually bring in a younger member of the family who translates. We don't have any problems with needing translators here. And that's just a geographic thing. I think it would be a whole lot more difficult to do the assessment if English wasn't the primary language. (G16, NSW)  One example comes to mind where a family misunderstood and thought that they had to go in for a  two to three hour assessment, which they've obviously misread that, that obviously occurs, if there's an issue and after 2 years they get the assessment, but there was there just it wasn't clear. And for them, at least for this particular family. And so there was it was very much “Oh, no, I'm not doing that. I don't have time for that.” And so I think, again, it comes back to time, a lot of time was required on both our part to explain things, and on the parents part to actually read all the information (G34, Victoria)  We have predominantly Middle Eastern, but we do have a lot of Asians and South American backgrounds and a lot of refugees. So sometimes, it may be a bit difficult to screen, because I don't speak another language. So sometimes bit difficult to identify children with autism. Sometimes parents can't voice their concerns as well. So sometimes there might be an issue like that. Also, you know, if the kids speak two different languages. A lot of parents sometimes put the speech delays into being that they're speaking two languages and they might not identify that as a problem. (G05, NSW) | |
| 4.4 |  | Family financial and socioeconomic circumstances | Finance, very very important. To get the NDIS approved they need a very comprehensive psychologist …psychiatric or paediatrician plus as follow up review. And until it wasn't approved, they won't get any funds. So they are really are … most of them are really under the pressure to get finance. I will do the care plan as well because they can use the care plan to see the different allied health, only five sessions a year which is really nothing. (G40, Victoria)  We have more healthcare card holders. And I know this because I get regular government type things, you know, where they tell you you're doing this, you've got such and such a population. And these are the number of people who are on health care card holders. And we are consistently higher than others for healthcare card holders. Bear in mind also that in terms of where the high birth rates were now, this is a few years old, but I think number one, birth rate is high at [suburb]. And the middle class is in [suburb]. And more around this area as well for the refugees the Asian community, the Korean community in [suburb]. There's quite a few kids around here and they're not necessarily the best resource for kids though. This area is gentrifying. And in fact the poor are getting squeezed out by the middle class. So maybe life will get better in the future. There'll be more services available because those with money generally have better infrastructures. But at the present time, no, there's a problem for getting services. (G27, NSW) | |
| 4.5 |  | Lack of workforce/resource | Behavioural problems or learning difficulties is a bit more of a minefield, because to get into the paediatricians who specialize in that sort of area, often has long waits. And there isn't anybody  specifically in this area, or there might be one paediatrician in [suburb] that we use, but a lot of the others are over at [suburb] or city, [suburb], I mean, not geographically close is what I'm saying. And more of an issue for them to get to. (G16, NSW)  I certainly know, there's some types of allied health interventions that are almost inaccessible for children, because the waiting list is so long that, you know, my joke is by the time they access it, it's not much use if they've become adults. (G33, Victoria) | |
| 4.6 |  | Patient denial of (potential or actual) child’s diagnosis | So sometimes it can be the parents that will delay the diagnosis because they don't want to accept. In fact, even now poor mum was extremely stressed because the child's not having OT care, speech development. The father is still in denial too. So we've got a lot of that social issues because the father refuses to see their child may have some sort of autism, autism, and so is refusing to accept [name of child]'s care. (G26, NSW) | |
| 4.7 |  | Complex navigation of the health system | I think the other problem is the, the lack of knowledge about available resources in the community which is very large and I always struggled to know not only the funding systems available, if there are concerns for children and parents, but also where to access them, because you could almost treat the same issue in five different ways, depending on your background and experience and local resources. So, you know, some will go down the specialist pathway, some will go by the Allied Health pathway, some would go through other structured programs. And again, it comes down to me making a guess, or having past experience in terms of what, what pathway I'm going to choose at the same time with the parents, I think, which may not be necessarily the best outcome, because some, some, some ways of treating it, I'm sure are better than others. But again, that might be an access thing as well. (G33, Victoria) | |
| **5.0** | **ASP pathway-specific barriers** | | | |
| 5.1 |  | Lengthened screening time | Some of the drawbacks to any sort of research at this particular time, especially if you try and do an 18 months session, they were sort of asking us to do the computer data was it's a very busy, busy time because 18 months vaccinations are like 12 months, essentially it's three vaccines... So that is already a barrier because we doctors are a hundred percent making sure that the vaccines are given correctly, that documentation is done correctly. So it's a very stressful time for a GP. (G26, NSW)  I didn't realise that was going to take up so much time at the beginning. I thought it was just part of my routine 18 months consult. But in fact during that secondary screening [SACS] actually takes these 15 or 20 more minutes that I hadn't counted on and it's not in my appointment book either. So they come in for the 18 month check and they get booked with the nurse as well for the vaccinations, but then I've got to find another 15 or 20 minutes to do that screening as well, which I think would have been nice to, to know that that was the time commitment. And then we could I don't know, explain to the parents, maybe when they book, this is what we're going to do. So there was a little bit of that lack of coordination perhaps with our front desk staff and admin and our bookings that we made it a little bit clunky. (G22, NSW)  I think it was more time consuming than realised at the outset. So it took, whilst it might just be, you know, a 10 minute thing, once you've read everything, the reading of everything, talk for the parent on saying yes to a lot of time, so they had to read and digest and consent and all of that. And then once they got through that stage, at other part might have been quick, but it wasn't a quick do this, while we vaccinate your child… (G34, Victoria) |  |
| 5.2 |  | Technical implementation issues | It was a bit clunky at the beginning that it was hard to you know, they're meant to fill out the  questionnaire and then we get an email back straight away with the result, but sometimes they didn't come back. On another days, they did fix it up. So now it's coming back, but sometimes it doesn't come back immediately. And so there's a kind of gap in between, see one or two other patients, and then they come back in and then to do that secondary screening. (G22, NSW)  The entire technical system was not timely. It wasn't totally enough. It didn't flow sufficiently. (G27 , NSW)  And you know, you have to do that second part [of the survey], even though there's not a lot of questions, but I think it's just more the technical side of it. Sometimes the internet doesn't work for whatever reason or that's just what slows it down. (G24 , NSW) |  |
| **6.0** | **Overlapping recommendations for both pathways** | | | |
| 6.1 |  | Need to clarify the role of GPs | One of the things that absolutely annoys me about the NDIS, is the exclusion of GPs, in general, from lots of the process, even though we are often the ones who have recommended that we go for an NDIS application, for some reason, we’re often peripheral. While in fact, in helping parents understand how to ask for funding that's associated with goals for positive change, and we are in a position to understand what may help. (G43, Victoria)  I think it's important for GPs to be involved in that process. In terms of referrals. So, I mean, general practice with anyone is that, you know, was supposed to be the sort of that centre point that is that, so that, you know, everything is communicated through the GP. So that everything centralised, and there's one person who knows what's going on, across whoever's involved in my management of that patient. The tricky thing being is that you see a lot of kids with developmental concerns that don't make the diagnosis. And your hands are a little bit tied on how much I mean, you can, help from a general practice perspective. (G36, Victoria) | |
| 6.2 |  | Need for further training and education | I'm a very strong advocate in education for our medical students and also for GPs’ need to pass the fellowship exams. Yeah. So what I'd like, if it could be put in a syllabus about RACGP syllabus for paediatrics, a standard approach to looking at milestones and giving us a little bit more education. I've trained as a GP back in the ’90s, we didn't have that. Now we have the diploma of paediatrics, but not all doctors are going to do it. Yeah. I don't think I need to do it, but it'd be lovely if we could have updates on paediatrics milestones and maybe then put it in a way for young doctors coming through as part of the syllabus, paediatric milestones. I think that would be great, you know, if the research was done and then that way we could then have a standard thing and then it will prompt me as a supervisor to make sure that my registrar is competent in those things. (G26, NSW) | |
| 6.3 |  | Need for a comprehensive, streamlined process | They get given the iPad straight away, or they get given a QR code and they can do it on their own phone. Perhaps that'd be even better than passing a company iPad around and do it on their own phone. And then we get the results by the time they finished the check with the nurse immunisations and we can discuss this results with them. And then if there's a pathway that says, do this next, or recall the patient in two months, or we will contact the patient. I think that'd be a great, a great tool for us. (G22, NSW) | |
| 6.4 |  | Need for funding | I'm a very strong advocate in education for our medical students and also for GPs’ need to pass the fellowship exams. Yeah. So what I'd like, if it could be put in a syllabus about RACGP syllabus for paediatrics, a standard approach to looking at milestones and giving us a little bit more education. I've trained as a GP back in the ’90s, we didn't have that. Now we have the diploma of paediatrics, but not all doctors are going to do it. Yeah. I don't think I need to do it, but it'd be lovely if we could have updates on paediatrics milestones and maybe then put it in a way for young doctors coming through as part of the syllabus, paediatric milestones. I think that would be great, you know, if the research was done and then that way we could then have a standard thing and then it will prompt me as a supervisor to make sure that my registrar is competent in those things. (G26, NSW)  I think, I would just have to admit that I lack the skills, I feel I lack the skills. I couldn't tell a parent how to improve speech or, you know, it's just not something that I'm trained in and have any exposure to, really… I would pretty much refer and support, but to specifically provide some instruction on, I’d hesitate, because I haven’t had the training … with the unacceptable delays in time, and crucial time that, you know, we could be doing something, if we were upskilled and we're able to assist and put people in the right direction of what they can do while they're waiting. Absolutely. (G34, Victoria) | |
| 6.5 |  | Need for digital developmental screening | I think this is great, because it means that the patients can fill it out in the waiting room, they can come in with the information, sometimes it helps them to focus their ideas, because sometimes they've just had a bad day with the child or things be a bit hectic, and they don't focus on what the problems are. … But I think this sort of screening program where you've got a, an app or a way that the parent can put the information in, means that it's all written down for the GP. And it becomes much clearer. (G16, NSW) | |
| **7.0** | **ASP pathway-specific recommendation** | | | |
| 7.1 |  | In-clinic administration support | Also having sort of like a navigator person in the waiting room, absolutely. Anything to help take the burden off the doctors and the nurses would be ideal. (G32, NSW) |  |
| **8.0** | **SaU pathway-specific recommendation** | | | |
| 8.1 |  | Need for a quick-reference parental/caregiver information |  | And I think that's more of an issue for parents where it might be their first child, and it's like, well, you know, are things on track, is it something we need to be worried about and, you know, sometimes getting another source other than me to confirm what I'm saying can be sort of reassuring in that sort of context. (G33, Victoria) |
